# Predator biomass and vegetation influence the coastal distribution of threespine stickleback morphotypes

**DOI:** 10.1002/ece3.7993

**Published:** 2021-08-12

**Authors:** Casey L. Yanos, Eeke P. Haanstra, Fiona Colgan Carey, Sorsha A. Passmore, Johan S. Eklöf, Ulf Bergström, Joakim P. Hansen, Michael C. Fontaine, Martine E. Maan, Britas Klemens Eriksson

**Affiliations:** ^1^ Groningen Institute for Evolutionary Life‐Sciences GELIFES University of Groningen Groningen The Netherlands; ^2^ Department of Ecology, Environment and Plant Sciences Stockholm University Stockholm Sweden; ^3^ Department of Aquatic Resources Swedish University of Agricultural Sciences Uppsala Sweden; ^4^ Stockholm University Baltic Sea Centre Stockholm Sweden; ^5^ Unité Mixe de Recherche MIVEGEC et Centre de Recherche en Ecologie et Evolution de la Santé Centre IRD de Montpellier CNRS IRD 229 Université de Montpellier Montpellier France

**Keywords:** ecosystem perturbation, ecotypic divergence, habitat specialization, intraspecific variation, stickleback

## Abstract

Intraspecific niche differentiation can contribute to population persistence in changing environments. Following declines in large predatory fish, eutrophication, and climate change, there has been a major increase in the abundance of threespine stickleback (*Gasterosteus aculeatus*) in the Baltic Sea. Two morphotype groups with different levels of body armor—completely plated and incompletely plated—are common in coastal Baltic Sea habitats. The morphotypes are similar in shape, size, and other morphological characteristics and live as one apparently intermixed population. Variation in resource use between the groups could indicate a degree of niche segregation that could aid population persistence in the face of further environmental change. To assess whether morphotypes exhibit niche segregation associated with resource and/or habitat exploitation and predator avoidance, we conducted a field survey of stickleback morphotypes, and biotic and abiotic ecosystem structure, in two habitat types within shallow coastal bays in the Baltic Sea: deeper central waters and shallow near‐shore waters. In the deeper waters, the proportion of completely plated stickleback was greater in habitats with greater biomass of two piscivorous fish: perch (*Perca fluviatilis)* and pike (*Esox lucius*). In the shallow waters, the proportion of completely plated stickleback was greater in habitats with greater coverage of habitat‐forming vegetation. Our results suggest niche segregation between morphotypes, which may contribute to the continued success of stickleback in coastal Baltic Sea habitats.

## Graphical abstract

Following declines in large predatory fish, eutrophication, and climate change, there has been a major increase in the abundance of two morphotype groups of threespine stickleback (*Gasterosteus aculeatus*) in the Baltic Sea. In deeper coastal waters, the proportion of completely plated stickleback was greater in habitats with greater biomass of two piscivorous fish: perch (*Perca fluviatilis*) and pike (*Esox lucius*) and in shallow coastal waters. The proportion of completely plated stickleback was greater in habitats with greater percent coverage of habitat‐forming vegetation. Our results suggest niche segregation between morphotypes, which may contribute to the continued success of stickleback in coastal Baltic Sea habitats.

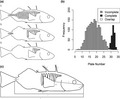

## 1 INTRODUCTION

Natural populations across the world are experiencing large‐scale changes in both biotic and abiotic conditions due to human activity. The probability of populations to persist in the face of environmental change is often related to niche variation within the population (Durell, 2000). For example, when predation is relaxed as a consequence of top predator loss, the resulting increased intraspecific competition between phenotypically similar individuals can promote divergence in resource exploitation (Zandonà et al., 2017). Such niche segregation may be followed by reduced gene flow between groups, reproductive isolation, and, if persisting for a long enough time, ecological speciation (Bush, 1994; Rice & Hostert, 1993; Smith, 1966). Niche expansion in response to environmental challenges implies that individuals can utilize resources beyond their typical ancestral range and can be indicative of populations' evolutionary potential (Bolnick et al., 2003). Therefore, documenting intraspecific niche variation may help to predict population persistence during environmental change.

In this study, we investigate niche segregation of threespine stickleback (*Gasterosteus aculeatus*) morphotypes that co‐occur in coastal bays in the Baltic Sea. The threespine stickleback (hereafter referred to as “stickleback”) is a small mesopredatory fish that shows rapid convergent evolution of functionally similar morphotypes across the Northern Hemisphere in response to environmental variation (McGee & Wainwright, 2013; McKinnon & Rundle, 2002). Populations are known to repeatedly diverge into morphotype groups that inhabit distinctly different habitats (Bentzen & McPhail, 1984; Bentzen et al., 1984; Schluter & McPhail, 1992). However, within a single population, sympatric morphological groups with different ecological behavior may also emerge (Harmon et al., 2009).

Stickleback have a broad distribution in freshwater, brackish, and marine habitats (Bell & Foster, 1994). In expanding stickleback populations, intraspecific competition often generates disruptive or divergent selection, particularly in areas with reduced predator populations (Bolnick, 2004; Schluter, 1994, 2010; Schluter & McPhail, 1992). Divergent adaptation in stickleback is largely mediated by standing genetic variation that enables the repeated divergence of distinct morphotypes (Colosimo et al., 2005; Jones et al., 2012; Schluter & Conte, 2009). The morphotypes are characterized by differences in feeding behavior, body size and shape, and the extent and pattern of body plating (reviewed in McKinnon & Rundle, 2002).

Lateral bony armor plates replace scales on stickleback, and the extent to which they cover the entire body defines the plate morphotype (Hagen & Gilbertson, 1973). Different morphotypes often occupy separate niches in terms of prey and habitat type, especially in relationship to the predation landscape (Bentzen et al., 1984; Des Roches et al., 2013; Schluter, 2010). Fully plated stickleback are thought to be better adapted to environments with higher fish predator density (Reimchen, 2000). Stickleback with fewer plates have greater maneuverability in terms of maximum velocity and displacement during fast‐start movement (Bergstrom, 2002; Reimchen, 1992). At the same time, high population densities may favor the evolution of adaptations that increase foraging efficiency (as seen in, e.g., guppies, Zandonà et al., 2017). Fast‐starts facilitate catching evasive prey and reduced plating could therefore be advantageous in areas with high intraspecific competition (Harper & Blake, 1988; Rand & Lauder, 1981).

**FIGURE 1:**
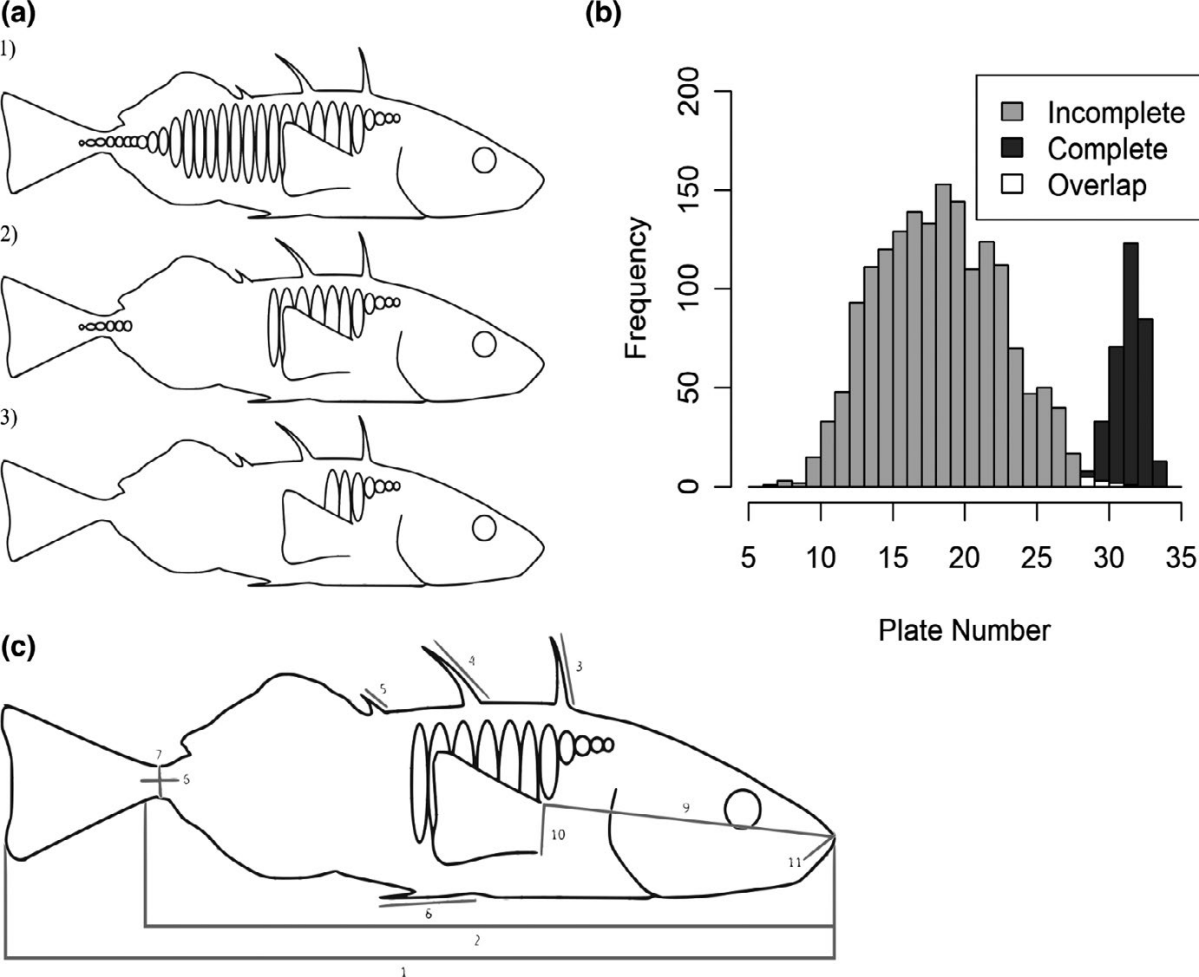
Plate patterns and frequencies of stickleback morphotypes commonly found in the Baltic Sea. (a) The three most common morphotypes in the Baltic Sea: (1) completely plated, (2) partially plated, and (3) low plated, (b) the frequency of stickleback morphotypes sampled across the central Swedish Baltic Coast in the spring of 2017, partially plated and low plated are grouped into an incompletely plated category, overlap represents plate counts that were present in both morphotype groups, and (c) Parameters measured for morphological analysis. (1) total length, (2) standard length, (3) length of first dorsal spine, (4) length of second dorsal spine, (5) length of third dorsal spine, (6) length of caudal peduncle, (7) depth of caudal peduncle, (8) length of pelvic spine, (9) head length, (10) width of pectoral fin base, (11) length of upper lip

In the Baltic Sea, a combination of overfishing, eutrophication, and climate change has restructured the food web which includes a dramatic increase in stickleback abundance (Alheit et al., 2005; Bergström et al., 2015; Casini et al., 2008; Eriksson et al., 2011; ICES, 2010; Ljunggren et al., 2010). Since the 1990s, adult stickleback have increased up to 45‐fold in offshore areas and up to 25‐fold in near‐shore areas along the Swedish coast (Bergström et al., 2015). In shallow bay habitats stickleback populations show a bimodal distribution in the number of lateral plates, with peaks at 15 and 29 plates, suggesting the presence of at least two different morphotypes (Aneer, 1974; Eriksson et al., 2021; Figure 1b). For the individuals that fall around the first peak (hereafter referred to as “incompletely plated”), plates are interrupted by one or more gaps, while in individuals that fall around the second peak (hereafter referred to as “completely plated”), plates run uninterrupted from immediately behind the operculum to the end of the caudal peduncle. Incompletely plated stickleback are typically separated into two morphotypes based on the presence or absence of caudal plates which form a keel. Those with caudal plates are designated as partially plated and those without as low plated. In the Swedish coastal populations, individuals without a keel are extremely rare. In two sampling seasons, Aneer (1974) found only two low‐plated stickleback (out of 1,791 individuals) and excluded them from analysis. In a sample of 560 stickleback caught during one season, Eriksson et al. (2021) did not observe any low‐plated individuals, but found a few intermediate phenotypes with a keel on only one side of their body. They therefore categorized all individuals with gaps in plating the width of two or more plates as incompletely plated, as we do here.

In the Baltic Sea, completely and incompletely plated stickleback live intermixed in an apparently single population; apart from the different plate numbers, they are morphologically similar. Complete and incomplete plating correspond to the plating patterns exhibited by stickleback in other geographic regions, such as the Pacific northwest of America and brackish water habitats in Norway (Hagen & Gilbertson, 1973; Østbye et al., 2018). While stickleback have been extensively studied in coastal Baltic Sea habitats for decades, few studies have considered the morphotypes in these populations and little is known about potential niche segregation (Byström et al., 2015; Candolin & Voigt, 1998; Jakubavičiūtė et al., 2018; Olsson et al., 2019; Saarinen & Candolin, 2020; Sieben et al., 2011). Recent evidence does indicate that while both morphotypes consume insects, zooplankton, and benthic invertebrates, completely plated stickleback consume more amphipods than incompletely plated stickleback. In addition, incompletely plated individuals make up a greater proportion of the stickleback population in areas of lower piscivorous fish biomass and greater benthic production, suggesting a trade‐off between predator defense and resource utilization (Eriksson et al., 2021). Exploitation of different resources by morphotypes could have substantial impacts on the Baltic food web, as stickleback populations dominate fish communities in many areas (Sieben et al., 2011; Staveley et al., 2020). For example, different relative abundances of Canadian lake benthic and limnetic stickleback used in a mesocosm experiment had different impacts on the biological community by modifying zooplankton species composition, algal biomass, primary productivity, dissolved organic content composition, and light transmission (Harmon et al., 2009).

The present study was designed to determine whether sympatric stickleback morphotypes exhibit niche segregation by occupying different microhabitats. We took samples from two habitat types in shallow coastal bays, assessing the relationship between (a) stickleback morphotype and fish community composition in deeper (≥1.0 m) central waters, and (b) stickleback morphotype composition and habitat variation in shallow (≤1.0 m) waters along the shoreline. In the Baltic Sea, shallow bays with high vegetation cover are characterized by a higher biomass of stickleback compared with nearby bays with less vegetation (Candolin & Selin, 2012; Saarinen & Candolin, 2020; Staveley et al., 2017, 2020). Within bays, stickleback are more abundant in macroalgal beds of the habitat‐forming brown algae *Fucus vesiculousus* (hereafter referred to as “*Fucus*”) compared with meadows of rooted, submerged vegetation and bare substrate (Gagnon et al., 2019). In those *Fucus* patches, the high density of stickleback may increase intraspecific competition for food or space, possibly leading to selection for competitive ability (foraging or territory defense), rather than antipredator traits. We therefore hypothesized that completely plated stickleback (a) make up a greater proportion of the stickleback community in bays where piscivorous fish are more abundant and (b) are less abundant in vegetated patches along the shoreline.

## 2 MATERIALS AND METHODS

### 2.1 Study area

The Baltic Sea is the world's second largest body of brackish water. It is almost completely enclosed by land, with the Danish Straits and Øresund being the only connections to the open waters of the North Sea. A strong salinity gradient is created by the input of freshwater from rivers and streams mainly in the north and saltwater from the Danish Straits in the southwest. Gradients in physical and chemical characteristics generate a diversity of habitats and biological communities (Rönnberg & Bonsdorff, 2004; Zettler et al., 2007). Diversity of habitat characteristics and biological communities can be very pronounced even at small spatial scales in the Baltic Sea due to the great topographic complexity of the coastal zone. For example, the archipelago region of the central Swedish Baltic Sea contains numerous bays (1–5 m depth) created by postglacial land uplift (Appelgren & Mattila, 2005; Hansen et al., 2008). The bays vary in their amount of water exchange and wave exposure from the open sea, creating a gradient in physical isolation (Hansen et al., 2008). However, the system is relatively open with fish migrating from the offshore open water of the Baltic Sea in the spring to mate in the bays before migrating back in the autumn (reviewed in Aro, 2002). Salinity in the bays ranges from around 1 to 7 practical salinity units (PSU). Vegetation composition consists of a mix of species with contrasting life histories, of marine or freshwater origin inhabiting hard or soft substrates. Open and more saline bays show higher proportions of hard substrates and perennial marine macroalgae (e.g., *Fucus*), while the most enclosed bays with organic rich sediments are dominated by rooted annual species of freshwater origin (e.g., *Chara* spp. and *Najas marina*) (Appelgren & Mattila, 2005; Hansen et al., 2008). Other common species in the bays are the freshwater species *Stuckenia pectinata*, *Potamogeton perfoliatus*, *Myriophyllum spicatum, Zannichellia palustris*, and the marine *Ruppia cirrhosa*. Some of these species grow drastically during the spring and summer and create variation in the habitats available for fish that migrate in during spring and summer (Berglund et al., 2003). The invertebrate community is mainly composed of gastropods, bivalves, insects, and crustaceans (Hansen et al., 2008).

### 2.2 Survey

**FIGURE 2:**
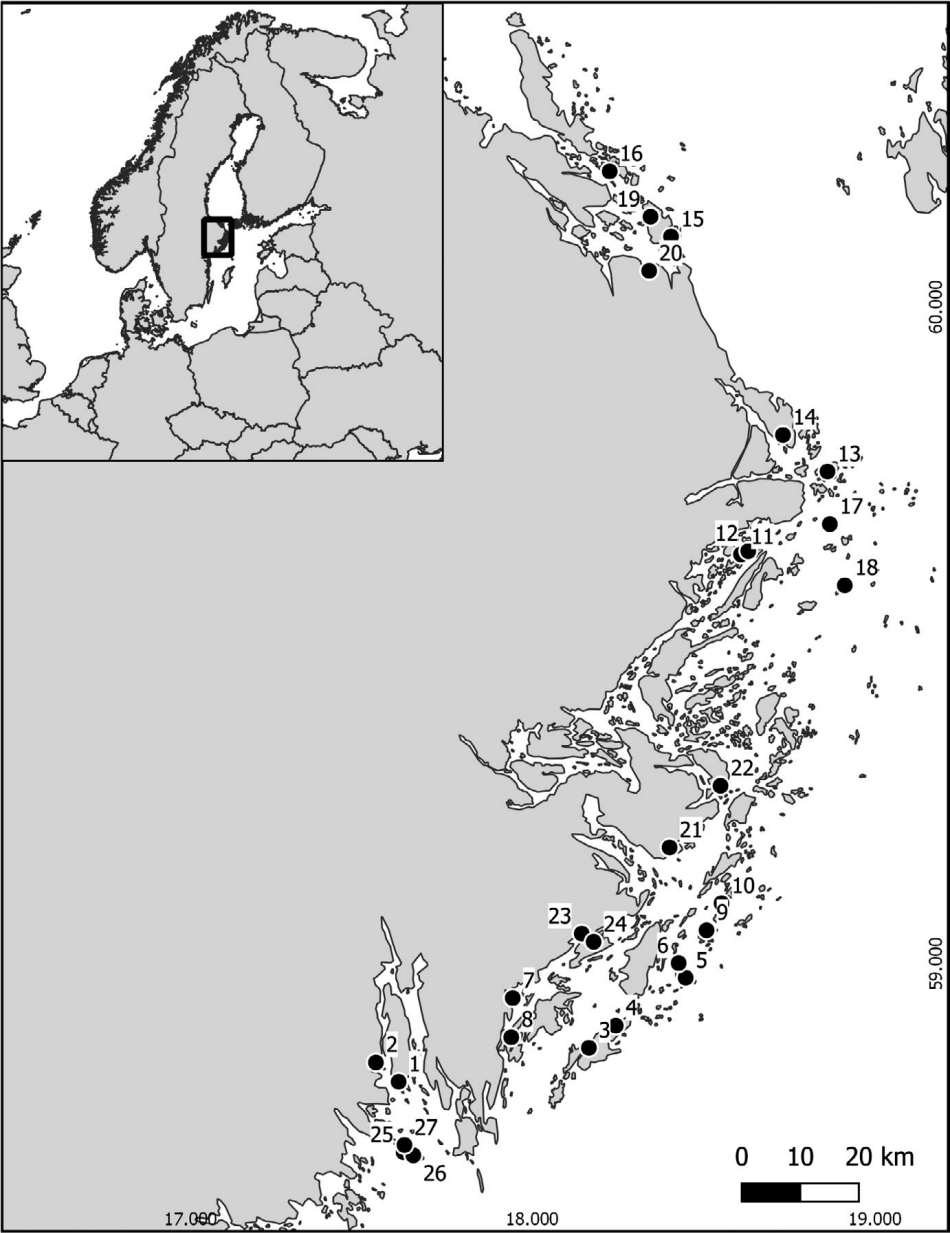
Map of sampling locations. Axis labels are latitude and longitude

We conducted field surveys from May to July 2017 at 27 bays along the central Swedish Baltic Sea coast (Figure 2). Sites were selected (in collaboration with the County Administrative Board of Stockholm) to represent a range of physical and biological characteristics, including habitats typical for both larger predatory fish and stickleback (Table S1). Twenty‐four of these sites were paired, with each pair containing two sites of similar physical characteristics, but one of the two was an MPA (Marine Protected Area) with a fishing ban from 1 April to 15 June (Table S1). We sampled two parts of each bay, the deeper water (≥1 m) in the center of the bay (hereafter referred to as “center‐of‐bay sampling”) and the shallow waters (≤1 m) along the shoreline (hereafter referred to as “shallow sampling”), for stickleback morphotype abundance and environmental properties. We chose these areas because they provide a snapshot of different biological communities within the bays. In the center‐of‐bay sampling, fish communities were sampled using gillnets, to obtain a broad representation of species and size classes. The shallow sampling, on the contrary, allowed us to sample more diverse vegetation as in these areas we find rocks with algae interspersed with reed and flowering vegetation, while in the deeper parts of the bay the vegetation is often more homogenous and the substrate is dominated by rock or mud.

#### 2.2.1 Center‐of‐bay sampling

To analyze variation in stickleback morphotype distribution in the central parts of the bays, we collected data on fish, vegetation, and substrate, as well as water temperature, salinity, and turbidity at 3–5 stations within each bay. Fish were sampled with 30 m long by 1.5 m high Nordic survey gillnets consisting of twelve 2.5 m long panels, each with a different mesh size: 43, 19.5, 6.25, 10, 55, 8, 12.5, 24, 15.5, 5, 35, and 29 mm knot‐to‐knot (European Union standardized method EN 14757:2005). Nets were placed at 1.5–3 m depth, following a stratified randomization. Three to five nets were set in each bay, depending on the surface area of the bay, for a total of 102 nets across 27 bays. The nets were set in the afternoon and retrieved the following morning. Fish were identified to species, measured for total length (cm), and counted. Stickleback were counted, but when more than 30 were caught only a haphazardly chosen sample of 30 individuals were measured (total length, mm) and preserved in 95% ethanol for later morphometric analysis. Total length was used to calculate biomass for all species following length/weight conversion factors from the Swedish National Database for Coastal Fish (http://www.slu.se/kul). Catch per unit effort (CPUE) was calculated as biomass per net night. At both ends of each net, vegetation surveys were conducted by a snorkeler who estimated the percent cover of all coarsely structured algae and plants, coverage of filamentous algae, average and maximum vegetation height, and substrate composition within a 5 m radius. Algae and plants were identified to the lowest taxonomic level possible. Substrate was categorized based on grain size of the sediment: mud, sand, gravel (2–20 mm), stone (20–200 mm), boulder (200–600 mm), large boulder (>600 mm), and bedrock. We also recorded water temperature and salinity with a multimeter and fluorescence and turbidity with an AquaFluor® fluorometer/turbidimeter (Turner Designs, California, USA) at each net.

#### 2.2.2 Shallow sampling

To analyze variation in shallow water along the shoreline, we collected data on fish, vegetation, and substrate. Fish were caught with 45 × 24 × 24 cm (length × width × height) minnow traps (KAYOBA, Skara, Sweden) with a mesh size of 3 mm and two 5.5 cm diameter round holes on each short side. To standardize the vegetation measurements around the trap, so that one measurement was always in front of the opening and one to the left and right, we closed off one hole on each trap with a cable tie. We set five traps for a total of 135 traps across 27 bays at 0.5–1 m depth. Traps were used instead of gillnets because of the dense vegetation and shallow depth range. Traps were deployed in the afternoon and retrieved the following morning. Traps were distributed such that they covered a gradient of vegetation cover. All fish caught were identified to species, measured for total length (mm), and counted. If more than 30 stickleback were caught in a trap they were all counted, but only a haphazardly chosen sample of 30 individuals were measured for total length (mm) and preserved in 95% ethanol for later morphometric analysis. At each trap, a vegetation survey was conducted by placing three 0.5 × 0.5 m frames within an estimated 5 m radius: one in front of the trap entrance and one on each long side of the trap. In each frame, we visually estimated the percent cover of all coarsely structured algae and plants, coverage of filamentous algae, average and maximum vegetation height, and substrate composition within the frame area. We chose to use a different method for the shallow vegetation survey than for the center‐of‐bay vegetation survey, because vegetation and substrate are more diverse in the shallow areas and using this method gave us a more detailed picture.

### 2.3 Morphometric analysis

We collected 2,085 stickleback for morphometric analysis, 1,672 from the center‐of‐bay sampling and 413 from the shallow sampling. Following the methods of Jones et al. (2012), we measured 11 length parameters using digital Vernier calipers (Figure 1c). To account for effects of body size, especially caused by age differences between individuals, we standardized all measurements to a standard length of 50 mm using the formula:

$${\hat{Y}}_{\mathrm{ijk}}={\bar{Y}}_{\mathrm{jk}}‐B_{\mathrm{jk}}\left(L_{\mathrm{ik}}‐50\right)$$

where *Y_ijk_* is the adjusted length of the *j*th variable of the *i*th individual from the *k*th population, ${\bar{Y}}_{\mathrm{jk}}$ is the mean of the *j*th variable length in the *k*th population, *B_jk_* is the coefficient of allometry for the *j*th variable on standard length within the *k*th population, *L_ik_* is the standard length of the *i*th individual in the *k*th population, and 50 is the standard length to which we adjusted all measurements (Thorpe, 1976; Lavin & McPhail, 1985; Hagen & Gilbertson, 1972). We compared morphometrics between morphotypes using Mann–Whitney *U* tests. We counted the number of lateral body plates on both sides of the fish, from immediately after the operculum to the end of the caudal peduncle. Morphotypes were categorized as follows: individuals with plates across the entire length of their body were categorized as completely plated, individuals with a gap in plates greater than or equal to the width of two body plates between the anterior plates and plates along the caudal peduncle were categorized as partially plated, and individuals with few anterior plates and no plates on the caudal peduncle were categorized as low plated (Figure 1a). In our sample, two fish were low plated on both sides of their body and four individuals were categorized as partially plated on one side of the body and low plated on the other side. We therefore pooled all partially and low‐plated stickleback into the single category of incompletely plated.

### 2.4 Data analysis

We analyzed the distribution of stickleback morphotypes using mixed‐effects logistic regression models with a binomial distribution and logit link function. We ran separate models for the center‐of‐bay and shallow data. In both cases, the dependent variable contained the numbers of completely plated and incompletely plated stickleback. For the center‐of‐bay data, we included net nested within bay as a random factor. We included the following fixed factors: coverage of habitat‐forming vegetation, piscivorous fish CPUE, maximum depth of the bay, wave exposure, bay location (inner, mid, or outer archipelago), relative latitudinal bay position (north, middle, or south), and bay topographic openness. We evaluated models with all possible combinations of predictor variables except variables which were collinear. Wave exposure was estimated from fetch and long‐term wind data using digital nautical charts and GIS methods, where refraction/diffraction effects are simulated by a spreading algorithm (Isæus, 2004; Sundblad et al., 2014). Bay location was categorized based on the relative shortest water distance to the open sea estimated by GIS methods (e.g., Eklöf et al., 2020). Bay topographic openness (*Ea*) was calculated using the formula:

Ea=100At/a

where *At* is the cross‐sectional area of the smallest connection to the sea, and *a* is bay surface area (e.g., Håkanson, 2008).

For the center‐of‐bay data, we only included sampling events where we caught at least 20 stickleback in the net which left us with 39 of the 101 nets. This threshold was chosen by first visually examining the proportion of completely plated individuals caught in the net plotted against the total number of stickleback caught in the bay, and then estimating where the variation in the proportion of completely plated individuals became consistent (Figure S1a). Continuous variables were on different scales so all were centered and scaled before analysis. We checked models for collinearity by calculating variance inflation factors (VIFs) of each predictor variable. Maximum depth of the bay, bay location, and relative bay position were collinear (VIF > 2) so they were included in separate models. When analyzing the shallow data, we included trap nested in bay as a random factor and coverage of habitat‐forming vegetation, *Fucus* presence, maximum depth of the bay, wave exposure, bay location, relative bay position, and bay topographic openness as fixed factors. We chose to include *Fucus* presence in the shallow data analysis as it is a perennial habitat‐forming species and hence is often the dominant vegetation habitat in the spring before annual or semiannual species have gained substantial biomass. All continuous variables were scaled and centered. Maximum depth of the bay, bay location, and relative bay position were collinear (VIF > 2) so they were included in separate models. For the shallow data, we ran two models, one where we only included data from traps with at least three stickleback (*n* = 36 traps out of the 135 traps in total) and another that only included data from traps with at least 10 stickleback (*n* = 14 traps). The thresholds were again chosen by visually examining the proportion of completely plated individuals caught in the trap plotted against the total number of stickleback caught and estimating where the variation in the proportion of completely plated individuals became consistent (Figure S1b). All analyses were conducted in R v. 3.5.1 (R Core Team, 2019). The models were run using the *lme4* package (Bates et al., 2015), and model selection was run based on the Akaike's information criterion corrected for small sample size (AICc) with the *MuMIn* package (Barton, 2019). Model assumptions were checked using functions in base R as well as the *DHARMa* package (Hartig, 2020).

## 3 RESULTS

We observed a wide range of variation in piscivore biomass, vegetation coverage, stickleback abundance, and stickleback morphotype relative abundance (Table S2). Of the 2,085 stickleback collected, we morphotyped 2,038 individuals (the rest were damaged during storage and transportation). We categorized 333 (16%) as completely plated and 1,705 (84%) as incompletely plated (two of which were low plated). Plate counts displayed a binomial distribution, corresponding to the completely plated and incompletely plated morphotype groups (Figure 1b). Most morphometric parameters, including standard length, did not differ between morphotype groups (*p* > .05, Table S3). The exceptions were length of the second and third dorsal spines and caudal peduncle depth which were all greater for incompletely than completely plated stickleback (*p* < .05, Table S3).

### 3.1 Center‐of‐bay microhabitat segregation

**FIGURE 3:**
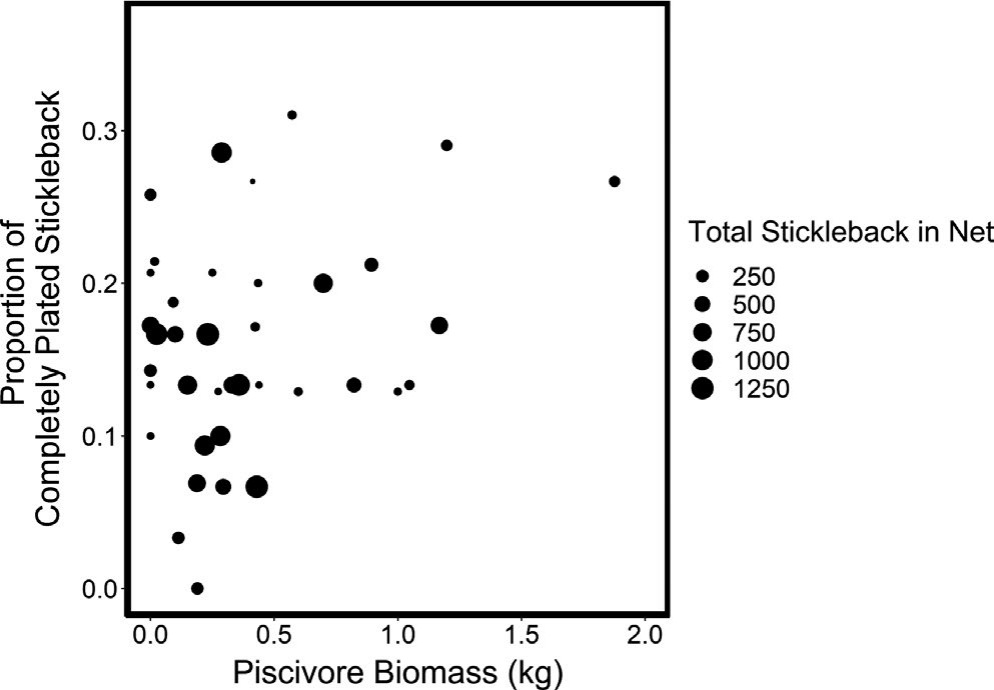
Relationship between piscivore biomass and proportion of completely plated stickleback from center‐of‐bay sampling from nets with at least 20 stickleback. Symbol sizes represent the total number of stickleback caught in each net

All models had similar AICc values (AICc = 186.1, 186.6, and 188.5 for the models constructed with maximum depth of the bay, relative latitudinal bay position, and bay location, respectively) and showed the same relationship between stickleback morphotype abundance and predictor variables. We focus on the model with the lowest AICc, which included maximum depth of the bay. Morphotype relative abundance was best predicted by piscivore biomass: Bays with greater piscivore biomass had higher proportions of completely plated stickleback (*R* ^2^ = 0.21, *p* < .01, Figure 3, Table S4). At low piscivore biomass, the entire range of proportions of completely plated stickleback was present. At higher piscivore biomass, the lower range of proportions of completely plated stickleback disappeared, resulting in a narrow range of relatively high proportions of completely plated stickleback (Figure 3).

### 3.2 Shallow‐water microhabitat segregation

**FIGURE 4:**
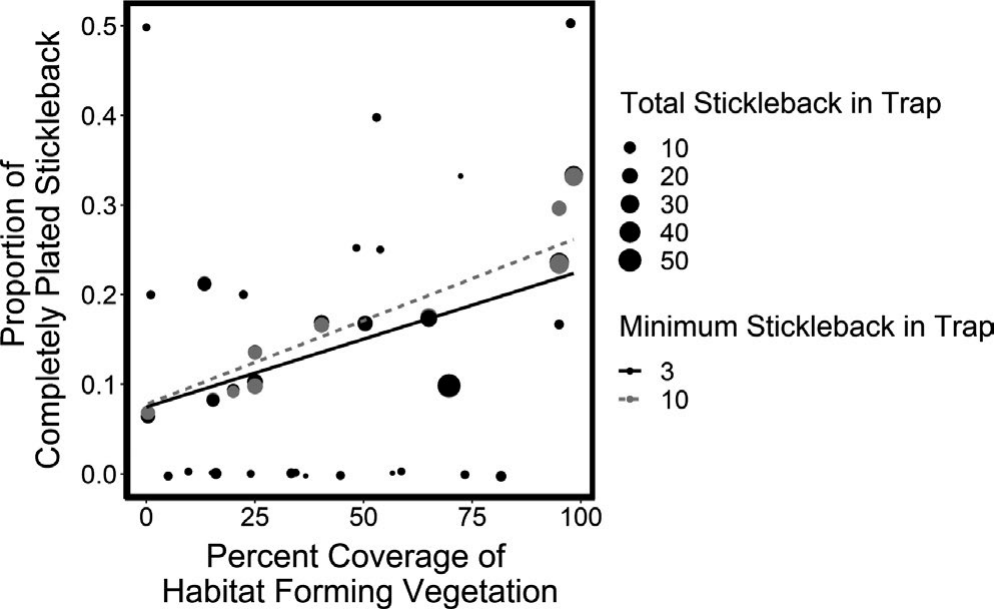
Relationship between percent coverage of habitat‐forming vegetation and proportion of completely plated stickleback from shallow sampling. Symbol sizes represent the total number of stickleback caught in each trap

All models had similar AICc values (AICc = 104.4, 104.6, and 104.8 for the models constructed with bay location, maximum depth of the bay, and relative bay position, respectively). All models showed the same relationship between stickleback morphotype abundance and predictor variables. We focus on the model with the lowest AICc, which included bay location. Morphotype relative abundance from the shallow‐water sampling was best predicted by vegetation coverage around the traps, at both thresholds (traps with at least three and 10 stickleback; Tables S5 and S6, respectively). In both models, there was a positive relationship between vegetation coverage and proportion of completely plated individuals (*R* ^2^ = 0.23, *p* < .01 and *R* ^2^ = 0.41, *p* < .01, respectively; Figure 4).

## 4 DISCUSSION

We investigated habitat use of two stickleback morphotypes in coastal Baltic Sea populations in order to determine the extent of intraspecific niche partitioning. We found that morphotype variation is associated with ecological differentiation along two axes of habitat variation. First, comparison of morphotype relative abundance in deeper central waters of the bays confirmed our hypothesis and earlier findings (Eriksson et al., 2021) that the proportion of completely plated stickleback morphotypes increases with increasing abundances of fish predators. Second, comparison of morphotype relative abundances in shallow waters along the shoreline shows that the completely plated morphotype is associated with a higher vegetation cover.

Disruptive selection which favors two morphotypes—one completely and one incompletely plated—is a recurrent pattern in freshwater stickleback populations (Marchinko et al., 2014; Zeller et al., 2012a). The completely plated morphotype is morphologically similar to the marine ancestor; it likely invaded from open water where it preferentially feeds on zooplankton and occupies the same habitat as large piscivorous fish (Taylor & McPhail, 2000). Body plating has been shown to serve as protection against predation and completely plated stickleback typically occupy niches dominated by predators (Reimchen, 1992, 1994, 1995). While full plating offers better protection against predation, low plating gives a higher body flexibility and possibly protects against insect predators, which may explain why less plated stickleback typically dominate in habitats with low piscivorous fish abundances (Reimchen, 1994; Zeller et al., 2012a). This is also attributed to variation in other morphological features. Comparisons of other stickleback populations show that dorsal spine length is positively correlated with piscivorous fish abundance and body plate number (Hagen & Gilbertson, 1972). It has also been suggested the dorsal spines and other bony structures are used by predatory insects to grasp onto stickleback (Reimchen, 1980). However, this spine reduction is in the number of dorsal spines which did not occur in our population (no individual had <3 dorsal spines) and an experimental study by Zeller et al. (2012b) showed no association between dorsal spine length and predation by insects. We did not collect data on predatory insects, so we cannot speak to this relationship. Despite a lack of reduction in number of dorsal spines, it still follows that the incompletely plated stickleback in our samples would have shorter spines as they are associated with lower abundances of piscivorous fish. However, length of the second and third dorsal spine are longer in the incompletely than completely plated stickleback and therefore do not follow the expected pattern. Caudal peduncle depth has also been shown to vary between morphotypes, possibly linked to habitat use. Freshwater stickleback typically have fewer plates than anadromous stickleback and stickleback with fewer plates have been shown to have better fast‐start performance (Bergstrom, 2002; Taylor and McPhail (1986). Freshwater stickleback occupy densely vegetated areas where their predators mainly strike but do not pursue them which makes fast‐start important to survival. We found caudal peduncles to be deeper in incompletely than completely plated stickleback, following the idea that fish which occupy areas with less piscivorous fish predators have better fast‐start performance, although this needs to be explicitly tested in our population.

Our results appear consistent with the overall pattern of greater abundances of stickleback with fewer plates in areas with less piscivorous fish predators. However, incompletely plated stickleback dominated all bays; the percentage of incompletely plated stickleback in the 27 bays never dropped below 60%, even at high piscivorous fish biomass (Table S2). This may be due to the low overall piscivorous fish biomass across the bays that we sampled and the dichotomy of piscivore‐ or stickleback‐dominated bays; in bays with piscivorous fish biomass above 2 kg, there were often few or no stickleback at all (Figure S2). Our study therefore may not have included the threshold of high piscivore biomass where completely plated stickleback would dominate (i.e., make up ≥50% of the stickleback community). The morphotype composition could also be influenced by the open sea fish community. Predation pressure is much lower in the open sea where stickleback spend the late autumn to early spring months and this may influence the morphotype composition of the entire population (Bergström et al., 2015; Eriksson et al., 2011). This could mean that regardless of the fish community in the coastal bays, completely plated stickleback will always make up a relatively low percentage of the stickleback community. Nevertheless, we observed an association between higher piscivore biomass and the relative abundance of completely plated stickleback in the range of data that we did have, suggesting that there is some association between piscivore and stickleback morphotype abundance.

In addition to piscivore abundance, or as an alternative explanation for the observed pattern, morphotype distribution may be influenced by abiotic environmental variables. Previous studies have suggested that completely plated stickleback tolerate higher salinities than incompletely plated stickleback and that across the stickleback's European range, higher abundances of completely plated individuals are observed in areas with higher salinity (Heuts, 1947). However, salinity hardly differed between our sampling locations (4.8–6.3 PSU; Table S1). Winter temperature has also been shown to influence morphotype distribution, with completely plated individuals tolerating lower temperatures than incompletely plated individuals (Smith et al., 2020). We did not include winter temperature in our analysis, but the temperature at the time of sampling did not affect morphotype abundance. Furthermore, temperature variation between our sites was mostly due to temporal variation (water temperatures were low during early spring sampling) while spatial variation in temperature was minimal (Tables S1 and S2). Lower pH and ion concentrations have also been associated with lower plate numbers (Bourgeois et al., 1994; Smith et al., 2020; Spence et al., 2013), but we did not measure these.

In shallow‐water sampling sites, we found a higher proportion of completely plated stickleback in microhabitats with high densities of habitat‐forming vegetation. This supports the idea that stickleback morphotypes segregate across microhabitats, but goes against a well‐established pattern in stickleback ecology: That incompletely plated stickleback are more flexible in their movements and therefore better able to maneuver through dense vegetation, which is what we based our hypothesis on (Bjærke et al., 2010; Leinonen et al., 2011; Reimchen, 1992). However, Bjærke et al. (2010) found that differences in spatial distribution may be related not just to plating but to overall body shape and showed that the largest differences in adaptation to different habitats were between low and completely plated stickleback while partially and completely plated stickleback were very similar. Our samples of incompletely plated stickleback mainly consist of partially plated individuals, and our morphometrics analysis showed that overall body shape varied little between incompletely and completely plated stickleback. Spatial distribution can also be related to foraging behavior. Stickleback with fewer plates have been shown to consume more benthic invertebrates while those with more plates consume more zooplankton (Bjærke et al., 2010; Larson & McIntire, 1993). Again, Bjærke et al. (2010) found the most pronounced dietary differences between low and completely plated stickleback. Larson and McIntire (1993) studied a population composed of morphotypes with no to few (<5) plates and still found pronounced differences in consumption of benthic and limnetic prey between individuals with different numbers of plates. In Swedish coastal bays similar to those in this study, completely plated stickleback were found to consume more amphipods than incompletely plated stickleback (Eriksson et al., 2021). The results of our study may therefore suggest that microhabitat segregation in shallow waters is based on feeding preference. In morphotype pairs outside the Baltic Sea, a tendency to eat more amphipods is associated with densely vegetated benthic areas (Bentzen & McPhail, 1984; Des Roches et al., 2013; Harmon et al., 2009). In coastal bays, amphipods are abundant in dense vegetation, particularly where there is *Fucus* (Schagerström et al., 2014). Our results therefore suggest that feeding preference may drive the association with dense vegetation as most patches of vegetation had some *Fucus* present.

While we hypothesize that differences in feeding preferences are linked to microhabitat segregation, we do not know to what extent it drives evolution. In other study systems, incompletely plated morphotypes have evolved from the marine fully plated ancestor (Taylor & McPhail, 2000). The partially plated morphotype may therefore be adapted to lower salinity habitats which could make them more successful in areas with more freshwater plants than *Fucus* where they feed on insects rather than marine amphipods (Heuts, 1947; Smith et al., 2020). This is, however, conjecture and cannot be tested with our data. To begin testing these hypotheses, feeding preferences must be examined. For this, morphometrics of feeding structures and stomach content data of the different morphotypes are needed. Gape size, suction strength, and gill raker length and number have all been shown to differ between stickleback with different diets (McGee & Wainwright, 2013; Robinson, 2000; Schluter & McPhail, 1992). We did not collect such data, with the exception of jaw length which did not differ between our morphotypes. Alternatively, our results could be driven, similar to the center‐of‐bay results, by the piscivorous fish community as there is typically higher piscivorous fish biomass in densely vegetated areas (Eklöv, 1997). However, sampling with minnow traps does not give us information on the piscivorous fish community (we caught a single perch and no pike in the traps). Perch are caught more effectively with gillnets and pike with rod and reel fishing. In the shallow sampling, we chose to use traps because they allowed fine‐scale microhabitat sampling, which would not be possible with the other methods.

One potential caveat of our study is that we collected stickleback during the mating season (Snickars et al., 2009). In some areas of the world, stickleback morphotypes have different nesting site preferences, where one morphotype prefers to nest in dense vegetation and the other on sand and gravel; however, no previous studies have examined nesting site preference in the Baltic Sea morphotypes (Candolin, 2004; Southcott et al., 2013). The segregation we see may therefore reflect not only the differences in predator avoidance and/or foraging strategy, but also nesting site preference. Thus, it may over‐ or underestimate the effects of predators and vegetation. Future studies should look at nest site preference and characterize (micro)habitat segregation throughout the year.

High abundance of stickleback has been shown to strongly impact lower trophic levels in the Baltic, generating trophic cascades that benefit filamentous algae, in both controlled experiments and comparative field surveys (Candolin et al., 2016; Donadi et al., 2017). Habitat segregation between the two morphotypes could intensify these ecosystem effects in at least two ways. First, it may simply allow more sticklebacks to coexist. Second, it may broaden their impact by affecting a wider range of prey groups. For example, completely plated stickleback may deplete algal grazer populations while incompletely plated stickleback deplete zooplankton populations. These hypotheses require further study, with possible implications for ecosystem management and restoration.

Here, we have presented a snapshot of habitat use during spring by two stickleback morphotypes in the Baltic Sea. The morphotypes are unevenly distributed across microhabitats. Combined with previously documented differences in diet (Eriksson et al., 2021), this suggests niche partitioning. In other ecosystems, stickleback are well known for rapid adaptive evolution (Colosimo et al., 2005; Kitano et al., 2008). Our study is notable because coastal bays in the Baltic Sea are highly connected to the open sea, and the different morphotypes seem to be fully overlapping in geographic distribution. Other sympatric morphotypes differ noticeably in morphology and utilize different spawning sites (i.e., different parts of a watershed or river) and thus have some restrictions to gene flow (Kume et al., 2010; Marques et al., 2016). Here, we show that even in an open system with constant multidirectional migration where morphotypes do not exhibit strong morphological differentiation other than plate number, there is affinity for different microhabitats. The slight habitat segregation may indicate ongoing directional evolutionary change (Bolnick et al., 2003; Rice & Hostert, 1993; Smith, 1966). Already before the recent increase in stickleback numbers, multiple plate morphotypes were present in coastal Baltic Sea habitats (Aneer, 1974). Thus, the presence of multiple morphotypes is not a recent phenomenon. However, there are no historical data on morphotype frequencies, resource preference, or spatial segregation of morphotypes. Future studies should place more emphasis on understanding the mechanisms driving this habitat segregation as it will help elucidate the overall response of the Baltic Sea ecosystem to ongoing and projected change.

## CONFLICT OF INTEREST

The authors declare no conflict of interest.

## AUTHOR CONTRIBUTIONS

**Casey L. Yanos:** Conceptualization (lead); data curation (lead); formal analysis (lead); investigation (lead); methodology (equal); project administration (equal); visualization (lead); writing‐original draft (lead); writing‐review & editing (lead). **Eeke P. Haanstra:** Investigation (supporting); writing‐review & editing (supporting). **Fiona Colgan Carey:** Investigation (supporting); writing‐review & editing (supporting). **Sorsha A. Passmore:** Investigation (supporting); writing‐review & editing (supporting). **Johan S. Eklöf:** Conceptualization (lead); funding acquisition (equal); investigation (supporting); methodology (equal); project administration (equal); supervision (lead); writing‐review & editing (supporting). **Ulf Bergström:** Conceptualization (supporting); investigation (supporting); methodology (equal); project administration (equal); writing‐review & editing (supporting). **Joakim P. Hansen:** Conceptualization (supporting); investigation (supporting); methodology (equal); project administration (equal); writing‐review & editing (supporting). **Michael C. Fontaine:** Conceptualization (supporting); funding acquisition (equal); writing‐review & editing (supporting). **Martine E. Maan:** Conceptualization (supporting); funding acquisition (equal); supervision (supporting); writing‐original draft (supporting); writing‐review & editing (supporting). **Britas Klemens Eriksson:** Conceptualization (lead); data curation (supporting); formal analysis (supporting); funding acquisition (equal); investigation (lead); methodology (equal); project administration (equal); supervision (lead); writing‐original draft (supporting); writing‐review & editing (supporting).

## Supporting information

Supplementary Material

Data S1

## Data Availability

Data are available as supplementary material.
