# Supplementary material for: Predator biomass and vegetation influence the coastal distribution of threespine stickleback morphotypes
Source: Ecol Evol. 2021 Aug 12;11(18):12485–96. doi: 10.1002/ece3.7993 (PMC8462182; doi:10.1002/ece3.7993)
Supplement: Supplementary file 1 — Supplementary Material [file ECE3-11-12485-s001.docx]

**Supplementary material**

**Appendix 1**

**
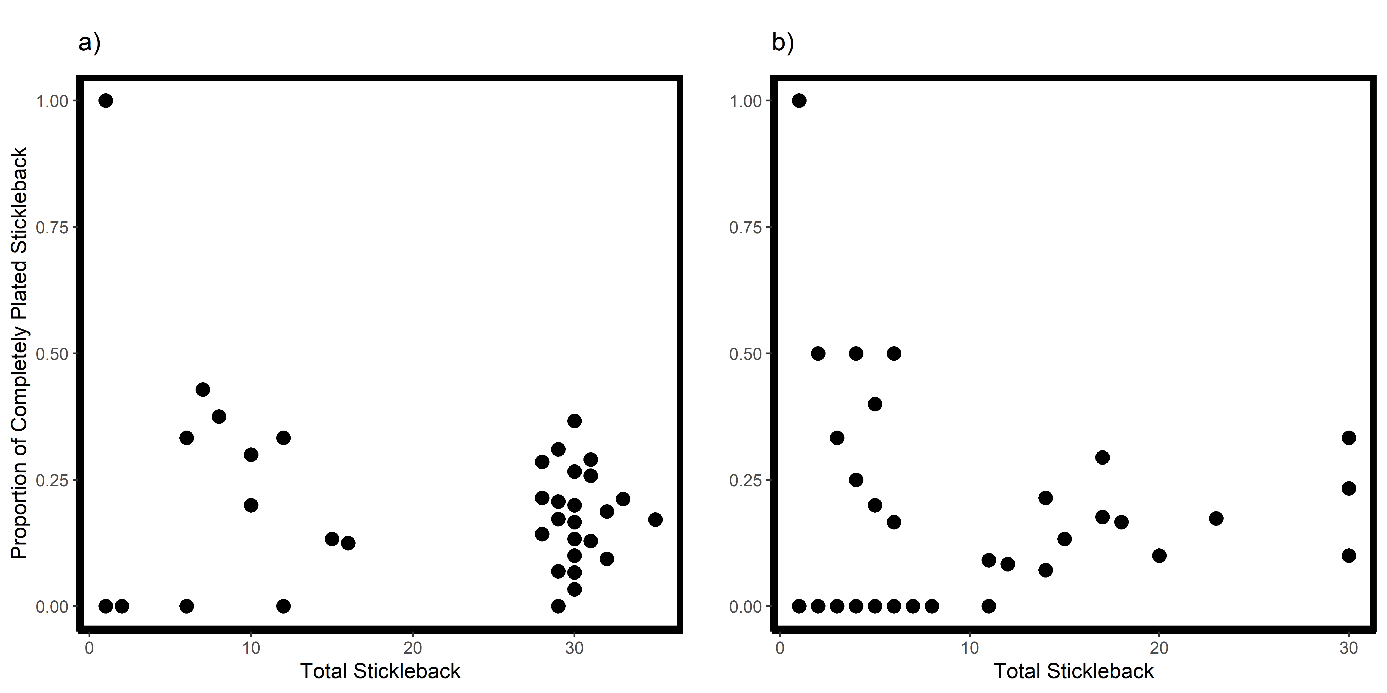
**

Figure S1. Proportion of completely plated stickleback in the catches for a) center-of-bay sampling and b) shallow sampling, against the total number of stickleback in each catch.


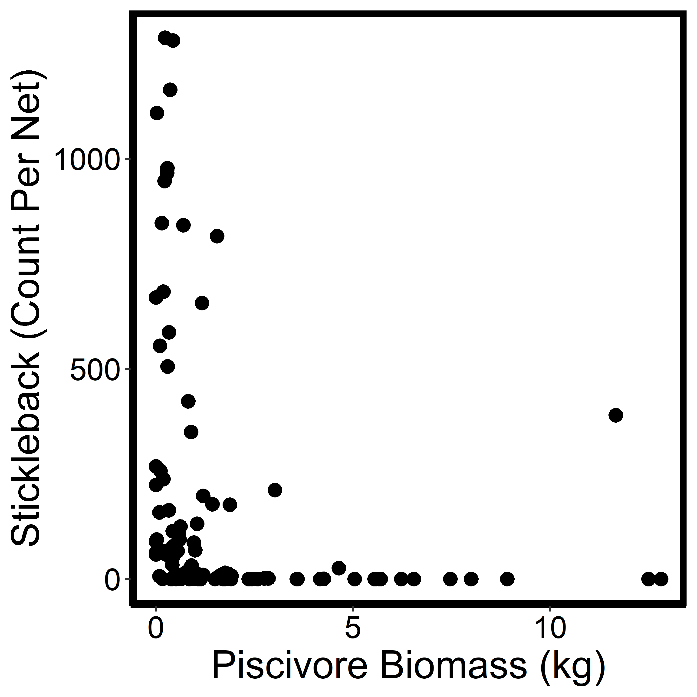


Figure S2. Relationship between piscivore biomass (in kg) and total stickleback count from center-of-bay sampling.

Table S1. Locations and physical characteristics of sites sampled in 2017. The table includes Site Number which corresponds to Figure 2 of the main text, the Swedish name of the site (Site Name), whether the site is a marine protected area (MPA) or not (Ref) (Status), which two bays were paired up (Bay Pair), relative latitudinal bay position within the archipelago (Position), location within the archipelago relative to the mainland (Location), the deepest part of the bay in meters (Maximum Depth), the distance in meters from the baseline which is defined as the start of territorial waters at the outermost islands of the archipelago (Distance From Baseline), surface wave exposure calculated with a 25 m resolution wave model (Wave Exposure), surface area (Area), the smallest opening connected to the sea (Opening Area), a dimensionless measure of the bay topographic openness (Openness), mean time water spends in the bay (Retention Time), mean salinity (Salinity), mean water temperature (Temperature), and the days the bay was sampled (Sampling Days).

| Site Number | Site Name | Latitude | Longitude | Status | Bay Pair | Position | Location | Maximum Depth  (m) | Distance From Baseline (m) | Wave Exposure (m^2^/s) | Area  (km^2^) | Opening Area (m^2^) | Openness | Retention Time (days) | Salinity (PSU) | Temperature (°C) | Sampling Days |
| --- | --- | --- | --- | --- | --- | --- | --- | --- | --- | --- | --- | --- | --- | --- | --- | --- | --- |
| 1 | Svalhagsviken | 58.92144 | 17.66088 | MPA | 1 | South | Inner | 5.7 | 30576 | 3644 | 1.462 | 2704 | 0.185 | 5.1 | 6.2 | 15.4 | 26/05/2017 : 27/05/2017 |
| 2 | Häggnäsviken | 58.95439 | 17.59264 | Ref | 1 | South | Inner | 5.2 | 35789 | 6839 | 0.748 | 2181 | 0.291 | 3.2 | 6.3 | 12.6 | 25/05/2017 : 26/05/2017 |
| 3 | Ryss-sundet/Byviken | 58.93603 | 18.22834 | MPA | 2 | South | Middle | 3.5 | 11188 | 1899 | 0.443 | 509 | 0.115 | 7.6 | 5.9 | 17.8 | 29/05/2019 : 30/05/2017 |
| 4 | Kyrkviken/Utö | 58.9629 | 18.30283 | Ref | 2 | South | Middle | 6 | 14399 | 2600 | 0.360 | 1183 | 0.328 | 2.7 | 5.9 | 13.1 | 30/05/2019 : 31/05/2017 |
| 5 | Ängsöfladen | 59.02605 | 18.53844 | MPA | 4 | South | Outer | 6 | 7497 | 7953 | 0.115 | 271 | 0.237 | 4.0 | 5.8 | 6.8 | 08/05/2017 : 09/05/2017 |
| 6 | Mörkviken/ Fjärdlång | 59.0504 | 18.52254 | Ref | 4 | South | Outer | 6 | 12409 | 2211 | 0.087 | 310 | 0.358 | 2.5 | 5.8 | 6.4 | 09/05/2017: 10/05/2017 |
| 7 | Hansviken | 59.02753 | 18.02307 | MPA | 5 | South | Inner | 2.4 | 27752 | 2445 | 0.035 | 117 | 0.331 | 2.7 | 5.9 | 7.9 | 10/05/2017 : 11/05/2017 |
| 8 | Söderängsviken/ Herrön | 58.9719 | 18.00424 | Ref | 5 | South | Inner | 4.8 | 25670 | 3053 | 0.073 | 217 | 0.297 | 3.1 | 5.9 | 8.1 | 11/05/2017 : 12/05/2017 |
| 9 | Norra fladen/Villinge | 59.09632 | 18.61324 | MPA | 6 | Middle | Outer | 5 | 10511 | 3449 | 0.433 | 169 | 0.039 | 13.9 | 5.9 | 9.2 | 11/05/2017 : 12/05/2017 |
| 10 | Fladorna på Östra Jungfruskär | 59.13937 | 18.68248 | Ref | 6 | Middle | Outer | 2.4 | 12338 | 1820 | 0.259 | 176 | 0.068 | 10.6 | 5.9 | 7.6 | 10/05/2017 : 11/05/2017 |
| 11 | Söderfladen | 59.66354 | 18.86633 | MPA | 8 | North | Inner | 2.7 | 32473 | 1429 | 0.072 | 12 | 0.017 | 18.7 | 5.5 | 16.2 | 19/05/2017 : 20/05/2017 |
| 12 | Eknöviken | 59.66731 | 18.88962 | Ref | 8 | North | Inner | 3 | 29600 | 1259 | 0.079 | 1 | 0.001 | 29.2 | 5.5 | 17.4 | 20/05/2017 : 21/05/2017 |
| 13 | Gisslingöfladen | 59.77396 | 19.16514 | MPA | 9 | North | Middle | 2.2 | 9877 | 1482 | 0.092 | 39 | 0.042 | 13.4 | 5.4 | 10.5 | 17/05/2017 : 18/05/2017 |
| 14 | Tofladen/  Gropaviken | 59.83884 | 19.04144 | Ref | 9 | North | Middle | 6 | 12909 | 1547 | 0.129 | 354 | 0.27 | 3.4 | 5.5 | 11.6 | 18/05/2017 : 19/05/2017 |
| 15 | Tranviksfjärden | 60.16216 | 18.78119 | MPA | 10 | North | Outer | 3 | 6477 | 1432 | 0.156 | 10 | 0.006 | 23.3 | 5.1 | 15.1 | 22/05/2017 : 23/05/2017 |
| 16 | Söderöfjärden/  Sladdarön | 60.26954 | 18.62787 | Ref | 10 | North | Outer | 3.9 | 17139 | 1370 | 0.246 | 258 | 0.105 | 8.1 | 5.1 | 12.6 | 23/05/2017 : 24/05/2017 |
| 17 | Sunden N Sundskär | 59.69214 | 19.14541 | MPA | 11 | North | Outer | 1 | 16819 | 1080 | 0.234 | 484 | 0.207 | 4.6 | 5.5 | 8.7 | 16/05/2017 : 17/05/2017 |
| 18 | Rödlöga/Högskär | 59.59636 | 19.16603 | Ref | 11 | North | Outer | 3 | 22247 | 1568 | 0.068 | 445 | 0.652 | 1.0 | 5.7 | 6.3 | 15/05/2017 : 16/05/2017 |
| 19 | Dalviken/  Bodafjärden | 60.20238 | 18.72852 | MPA | 7 | North | Middle | 6 | 13992 | 2372 | 0.254 | 676 | 0.266 | 3.5 | 5.0 | 11.5 | 23/05/2017 : 24/05/2017 |
| 20 | Rotholmaviken | 60.1162 | 18.69427 | Ref | 7 | North | Outer | 3 | 11840 | 3461 | 0.437 | 531 | 0.121 | 7.2 | 5.0 | 14.4 | 24/05/2017 : 25/05/2017 |
| 21 | Torpe-Infjärden, Björnöfjärden | 59.22687 | 18.54536 | MPA | 12 | Middle | Inner | 8.2 | 27154 | 2545 | 1.445 | 40 | 0.003 | 26.1 | 5.1 | 9.5 | 08/05/2017 : 09/05/2017 |
| 22 | Djuröviken | 59.31725 | 18.71796 | Ref | 12 | Middle | Inner | 5.1 | 31450 | 1841 | 0.182 | 13 | 0.007 | 22.8 | 4.8 | 9.0 | 09/05/2017 : 10/05/2017 |
| 23 | Lännåkersviken | 59.11518 | 18.24139 | MPA | 13 | Middle | Inner | 4.5 | 30628 | 3291 | 0.444 | 134 | 0.030 | 15.4 | 5.9 | 14.2 | 01/06/2017 : 02/06/2017 |
| 24 | Askviken | 59.09716 | 18.27829 | Ref | 13 | Middle | Inner | 4.7 | 28306 | 2673 | 0.339 | 39 | 0.012 | 20.6 | 5.8 | 14.8 | 31/05/2017 : 01/06/2017 |
| 25 | Idkroken | 58.81273 | 17.64653 | Ref | NA | South | Middle | 4.1 | 18959 | 42234 | 0.089 | 258 | 0.288 | 3.2 | 6.2 | 19.7 | 19/06/2017 : 20/06/2017 |
| 26 | Skutviken | 58.80534 | 17.67558 | Ref | NA | South | Middle | 3.9 | 17533 | 21772 | 0.035 | 213 | 0.612 | 1.1 | 6.1 | 14.6 | 21/06/2017 : 22/06/2017 |
| 27 | Hästdammsviken | 58.8224 | 17.65245 | Ref | NA | South | Middle | 3.2 | 20399 | 8247 | 0.015 | 517 | 3.390 | 0.0 | 6.2 | 16.5 | 23/06/2017 : 24/06/2017 |

Table S2. Stickleback abundance and resources surveyed which came out as significant predictors of morphotype relative abundance.

|  | Total Stickleback (Count) | | | | Morphotyped Stickleback (Count) | | | | Proportion Complete Morphs | | | | Coverage of Habitat Forming Vegetation (%) | | | | Fucus Presence | Piscivore Biomass (kg) | |
| --- | --- | --- | --- | --- | --- | --- | --- | --- | --- | --- | --- | --- | --- | --- | --- | --- | --- | --- | --- |
|  | Nets |  | Traps |  | Nets |  | Traps |  | Nets |  | Traps |  | Nets |  | Traps |  | Traps | Nets |  |
| Bay | Average | Range | Average | Range | Average | Range | Average | Range | Average | Range | Average | Range | Average | Range | Average | Range | # Traps | Average | Range |
| 1 | 7.80 | 1 - 15 | 0.00 | 0 - 0 | 7.80 | 1 - 15 | 0.00 | 0 - 0 | 0.13 | 0.00 - 0.30 | - | - | 13.10 | 5.00 - 42.50 | 98.67 | 80.00 - 116.67 | 5 | 1.39 | 0.56 - 2.76 |
| 2 | 292.20 | 56 - 684 | 0.00 | 0 - 0 | 30.80 | 30 - 32 | 0.00 | 0 - 0 | 0.13 | 0.03 - 0.22 | - | - | 65.00 | 33.00 - 95.00 | 87.40 | 67.67 - 104.67 | 5 | 0.34 | 0.19 - 0.55 |
| 3 | 97.50 | 0 - 390 | 7.20 | 1 - 18 | 7.75 | 0 - 30 | 6.60 | 1 - 17 | 0.18 | 0.00 - 0.37 | 0.04 | 0.00 - 0.18 | 22.13 | 8.00 - 56.00 | 62.07 | 18.00 - 106.67 | 3 | 9.43 | 5.55 - 12.50 |
| 4 | 777.25 | 657 - 966 | 13.40 | 4 - 23 | 30.00 | 30 - 30 | 13.40 | 4 - 23 | 0.14 | 0.10 - 0.17 | 0.07 | 0.00 - 0.17 | 82.88 | 78.00 - 86.50 | 45.20 | 26.00 - 78.33 | 5 | 0.75 | 0.00 - 1.55 |
| 5 | 207.00 | 53 - 506 | 0.20 | 0 - 1 | 30.33 | 30 - 31 | 0.20 | 0 - 1 | 0.10 | 0.07 - 0.13 | 0.00 | 0.00 - 0.00 | 15.33 | 5.50 - 28.00 | 3.27 | 0.00 - 7.33 | 2 | 0.24 | 0.00 - 0.44 |
| 6 | 63.33 | 58 - 69 | 4.20 | 1 - 15 | 31.00 | 30 - 32 | 3.80 | 1 - 14 | 0.16 | 0.13 - 0.21 | 0.11 | 0.00 - 0.50 | 13.00 | 2.00 - 32.50 | 9.47 | 0.33 - 36.67 | 5 | 0.42 | 0.00 - 1.00 |
| 7 | 2.67 | 0 - 7 | 0.00 | 0 - 0 | 2.67 | 0 - 7 | 0.00 | 0 - 0 | 0.71 | 0.43 - 1.00 | - | - | 8.67 | 5.50 - 10.50 | 5.47 | 0.00 - 20.00 | 0 | 0.37 | 0.09 - 0.65 |
| 8 | 0.33 | 0 - 1 | 0.00 | 0 - 0 | 0.33 | 0 - 1 | 0.00 | 0 - 0 | 0.0.00 | 0.00 - 0.00 | - | - | 2.17 | 0.50 - 5.00 | 14.33 | 0.00 - 30.00 | 1 | 1.05 | 0.17 - 2.58 |
| 9 | 208.20 | 159 - 268 | 2.40 | 1 - 5 | 30.40 | 29 - 33 | 2.20 | 1 - 5 | 0.12 | 0.00 - 0.19 | 0.00 | 0.00 - 0.00 | 50.40 | 3.00 - 97.00 | 83.73 | 11.00 - 1111.67 | 5 | 0.73 | 0.00 - 3.02 |
| 10 | 58.00 | 0 - 258 | 1.40 | 0 - 4 | 12.00 | 0 - 30 | 1.40 | 0 - 4 | 0.12 | 0.03 - 0.20 | 0.38 | 0.00 - 1.00 | 12.30 | 4.50 - 21.5 | 0.47 | 0.00 - 1.33 | 0 | 5.40 | 0.11 - 12.82 |
| 11 | 0.00 | 0 - 0 | 0.00 | 0 - 0 | 0.00 | 0 - 0 | 0.00 | 0 - 0 | - | - | - | - | 83.83 | 65.00 - 98.00 | 3.67 | 0.00 - 10.00 | 0 | 3.26 | 0.51 - 5.70 |
| 12 | 0.00 | 0 - 0 | 0.00 | 0 - 0 | 0.00 | 0 - 0 | 0.00 | 0 - 0 | - | - | - | - | 1.67 | 0.00 - 2.50 | 10.13 | 0.00 - 46.67 | 0 | 2.68 | 1.09 - 5.04 |
| 13 | 0.33 | 0 - 1 | 0.00 | 0 - 0 | 0.33 | 0 - 1 | 0.00 | 0 - 0 | 0.00 | 0.00 - 0.00 | - | - | 35.33 | 8.50 - 60.00 | 9.00 | 0.00 - 25.00 | 1 | 3.66 | 1.13 - 5.59 |
| 14 | 2.33 | 0 - 6 | 0.00 | 0 - 0 | 3.67 | 0 - 10 | 0.00 | 0 - 0 | 0.65 | 0.30 - 1.00 | - | - | 2.33 | 1.50 - 3.50 | 30.07 | 11.67 - 50.00 | 2 | 3.13 | 1.08 - 6.54 |
| 15 | 5.00 | 0 - 13 | 2.40 | 0 - 4 | 5.00 | 0 - 13 | 2.40 | 0 - 4 | 0.00 | 0.00 - 0.00 | 0.06 | 0.00 - 0.25 | 65.33 | 30.50 - 89.50 | 90.17 | 65.33 - 111.50 | 5 | 2.06 | 1.50 - 2.85 |
| 16 | 33.67 | 0 - 94 | 2.60 | 1 - 5 | 12.33 | 0 - 31 | 2.60 | 1 - 5 | 0.23 | 0.13 - 0.33 | 0.07 | 0.00 - 0.33 | 22.17 | 1.50 - 61.5 | 34.47 | 0.00 - 73.33 | 0 | 1.93 | 0.60 - 3.59 |
| 17 | 112.25 | 78 - 132 | 3.40 | 1 - 7 | 31.75 | 30 - 36 | 3.40 | 1 - 7 | 0.14 | 0.07 - 0.20 | 0.14 | 0.00 - 0.50 | 47.38 | 12.00 - 92.00 | 98.47 | 25.00 - 165.00 | 5 | 0.63 | 0.42 - 1.05 |
| 18 | 116.50 | 59 - 224 | 6.80 | 2 - 18 | 29.75 | 28 - 31 | 6.80 | 2 - 18 | 0.20 | 0.13 - 0.26 | 0.17 | 0.00 - 0.50 | 18.75 | 2.00 - 45.00 | 136.07 | 31.00 - 192.33 | 5 | 0.00 | 0.00 - 0.02 |
| 19 | 353.75 | 87 - 555 | 26.80 | 4 - 50 | 30.75 | 30 - 33 | 22.20 | 4 - 30 | 0.16 | 0.13 - 0.21 | 0.24 | 0.10 - 0.33 | 21.00 | 2.00 - 50.00 | 83.43 | 48.33 - 98.33 | 5 | 0.69 | 0.10 - 0.96 |
| 20 | 34.75 | 10 - 107 | 1.00 | 0 - 4 | 15.75 | 10. - 31 | 1.00 | 0 - 4 | 0.26 | 0.10 - 0.33 | 0.00 | 0.00 - 0.00 | 17.75 | 0.50 - 35.50 | 39.00 | 20.00 - 66.67 | 0 | 1.12 | 0.57 - 1.66 |
| 21 | 0.00 | 0 - 0 | 0.00 | 0 - 0 | 0.00 | 0 - 0 | 0.00 | 0 - 0 | - | - | - | - | 23.75 | 5.00 - 77.5 | 21.33 | 3.33 - 36.67 | 0 | 3.45 | 1.70 - 7.99 |
| 22 | 0.00 | 0 - 0 | 0.00 | 0 - 0 | 0.00 | 0 - 0 | 0.00 | 0 - 0 | - | - | - | - | 13.88 | 10.00 - 18.00 | 5.33 | 0.00 - 26.67 | 0 | 4.61 | 0.85 - 8.91 |
| 23 | 6.20 | 0 - 16 | 0.00 | 0 - 0 | 6.20 | 0 - 16 | 0.00 | 0 - 0 | 0.12 | 0.00 - 0.22 | - | - | 28.00 | 2.00 - 67.00 | 29.93 | 9.33 - 53.33 | 2 | 1.45 | 0.53 - 4.17 |
| 24 | 117.40 | 8 - 198 | 2.20 | 0 - 5 | 25.20 | 8 - 31 | 2.20 | 0 - 5 | 0.26 | 0.15 - 0.38 | 0.07 | 0.00 -0.20 | 23.10 | 11.50 - 39.50 | 43.87 | 4.00 - 100.67 | 4 | 2.21 | 1.20 - 4.64 |
| 25 | 810.33 | 34 - 1288 | 7.60 | 3 - 15 | 30.00 | 30 - 30 | 7.60 | 3 - 15 | 0.20 | 0.17 - 0.27 | 0.04 | 0.00 - 0.13 | 33.50 | 27.50 - 43.00 | 62.13 | 40.67 - 80.00 | 5 | 0.22 | 0.03 - 0.41 |
| 26 | 1068.33 | 947 - 1281 | 4.00 | 1 - 14 | 30.00 | 28 - 32 | 4.00 | 1 - 14 | 0.15 | 0.07 - 0.29 | 0.14 | 0.00 - 0.50 | 56.50 | 33.50 - 82.50 | 82.20 | 33.33 - 101.67 | 5 | 0.31 | 0.22 - 0.43 |
| 27 | 951.00 | 842 - 1164 | 1.40 | 0 - 5 | 30.00 | 30 - 30 | 14.00 | 0 - 5 | 0.16 | 0.13 - 0.20 | 0.13 | 0.00 - 0.40 | 38.17 | 16.50 - 65.50 | 113.13 | 106.33 - 122.67 | 5 | 0.40 | 0.15 - 0.70 |

Table S3. Comparison of morphometric characteristics between completely and incompletely-plated stickleback measured by Mann-Whitney U-tests; *, p<0.05.

|  | p |
| --- | --- |
| Total Length | 0.706 |
| First Dorsal Spine Length | 0.268 |
| Second Dorsal Spine Length | 0.034* |
| Third Dorsal Spine Length | 0.016* |
| Caudal Peduncle Length | 0.405 |
| Caudal Peduncle Depth | 0.017* |
| Pelvic Spine Length | 0.981 |
| Head Length | 0.462 |
| Pectoral Fin Base Width | 0.212 |
| Upper Lip/Jaw Length | 0.749 |

Table S4. Model selection for mixed effects logistic regression model predicting morphotype relative abundance of center-of-bay sampling showing the 10 best models as ranked by AICc. Model formula: Percent Completely Plated ~ Cover of Habitat Forming Vegetation + Openness + Maximum Depth + Piscivores + Wave Exposure + (1|Bay Number/Net).

| Model | AICc | Delta | Weight |
| --- | --- | --- | --- |
| Piscivores | 176.4 | 0.00 | 0.300 |
| Piscivores + Maximum Depth | 178.2 | 1.84 | 0.120 |
| Piscivores + Vegetation | 178.4 | 2.07 | 0.106 |
| Piscivores + Wave Exposure | 178.6 | 2.24 | 0.098 |
| Piscivores + Openness | 179.0 | 2.64 | 0.080 |
| Piscivores + Maximum Depth + Vegetation | 180.4 | 4.03 | 0.040 |
| Piscivores + Maximum Depth + Wave Exposure | 180.7 | 4.28 | 0.035 |
| Piscivores + Vegetation + Wave Exposure | 180.8 | 4.40 | 0.033 |
| Piscivores + Maximum Depth + Openness | 181.0 | 4.58 | 0.030 |
| Piscivores + Vegetation + Openness | 181.3 | 4.88 | 0.026 |

Table S5. Model selection for mixed effects logistic regression model predicting morphotype relative abundance of shallow sampling for traps with a cutoff of at least three stickleback per trap showing the 10 best models as ranked by AICc. Model Formula: Percent Completely Plated ~ Cover of Habitat Forming Vegetation + Fucus Presence + Openness + Wave Exposure + Bay Location + (1|Bay Number/Trap).

| Model | AICc | Delta | Weight |
| --- | --- | --- | --- |
| Vegetation | 94.9 | 0.00 | 0.296 |
| Vegetation + Openness | 96.4 | 1.52 | 0.138 |
| Vegetation + Fucus Presence | 97.3 | 2.42 | 0.088 |
| Vegetation + Bay Location | 97.5 | 2.64 | 0.079 |
| Vegetation + Wave Exposure | 97.6 | 2.69 | 0.077 |
| Vegetation + Openness + Fucus Presence | 98.3 | 3.47 | 0.052 |
| Vegetation + Openness + Wave Exposure | 99.2 | 4.29 | 0.035 |
| Vegetation + Openness + Bay Location | 99.2 | 4.36 | 0.033 |
| Intercept Only | 99.8 | 4.94 | 0.025 |
| Vegetation + Fucus Presence + Bay Location | 100.0 | 5.16 | 0.022 |

Table S6. Model selection for mixed effects logistic regression model predicting morphotype relative abundance of shallow sampling for traps with a cutoff of at least 10 stickleback per trap showing the 10 best models as ranked by AICc. Model Formula: Percent Completely Plated ~ Cover of Habitat Forming Vegetation + Fucus Presence + Openness + Wave Exposure + Bay Location + (1|Bay Number/Trap).

| Model | AICc | Delta | Weight |
| --- | --- | --- | --- |
| Vegetation | 53.4 | 0.00 | 0.608 |
| Intercept Only | 55.8 | 2.39 | 0.184 |
| Vegetation + Wave Exposure | 58.7 | 5.33 | 0.042 |
| Vegetation + Openness | 58.9 | 5.47 | 0.039 |
| Vegetation + Bay Location | 59.0 | 5.57 | 0.038 |
| Bay Location | 59.4 | 6.03 | 0.030 |
| Wave Exposure | 59.6 | 6.23 | 0.027 |
| Openness | 60.0 | 6.62 | 0.022 |
| Wave Exposure + Bay Location | 64.2 | 10.77 | 0.003 |
| Openness + Bay Location | 65.0 | 11.60 | 0.002 |
